# The Impact of Experiential Learning Using an Indoor Aquaponic System on Nutrition Students' Sustainable Food Knowledge and Behaviour

**DOI:** 10.1111/jhn.70103

**Published:** 2025-07-24

**Authors:** Isabelle Crowe, Karen E. Charlton, Anne‐Therese McMahon, Indiana Rhind, Katherine Kent

**Affiliations:** ^1^ School of Medical, Indigenous and Health Sciences, Faculty of Science, Medicine and Health University of Wollongong Wollongong New South Wales Australia

**Keywords:** experiential learning, knowledge, attitudes, practice, nutrition, sustainability, university

## Abstract

**Aims:**

Nutrition and dietetics students are future change agents for sustainable food systems, yet sustainability education remains limited in university curricula. This study evaluated the impact of an experiential learning activity using an indoor aquaponic system on students' knowledge, attitudes and behavioural intentions towards sustainable diets.

**Methods:**

An embedded mixed methods pre−post survey captured baseline data on students' sustainable diet knowledge, attitudes and behaviours, alongside diet quality (using the Australian Recommended Food Score). Students then participated in an interactive experiential learning activity involving an indoor aquaponic system. Post‐activity surveys assessed changes in knowledge and behaviour intentions, as well as perceptions of the learning experience. Quantitative data were analysed using chi‐square tests, regression and McNemar−Bowker tests; qualitative responses were thematically analysed.

**Results:**

At baseline, students (*n* = 58, 87.9% female) reported limited knowledge of local food systems, though most valued sustainable food practices. Students who placed high importance on sustainable diets were significantly more likely to engage in sustainable dietary behaviours. Diet quality was significantly higher among students who grew their own food, bought locally or purchased seasonal produce (all *p* < 0.05). Participation in the experiential learning activity significantly improved students' self‐reported knowledge of local food systems (*p* < 0.001) and increased their intentions towards buying locally‐grown (57.9% to 86.8%, *p* < 0.001) and growing food (36.8% to 78.9%, *p* < 0.001). Thematic analysis highlighted students' increased awareness of sustainability complexity, their role as nutrition professionals and the importance of curriculum integration.

**Conclusion:**

Experiential learning with an indoor aquaponic system enhanced students' knowledge and intentions related to sustainable diets. Students who engaged in sustainable behaviours had significantly higher diet quality, highlighting the potential dual benefit of curriculum integration. Embedding sustainability‐focused learning in nutrition curricula may better prepare students to lead change in food systems.

## Summary

- Sustainable food behaviours were linked to higher diet quality in nutrition and dietetics students.
- Experiential learning with an aquaponic system improved students' knowledge and intentions around sustainable diets.
- Embedding sustainability and systems thinking in curricula can better prepare students for practice.

## 1 Introduction

The rapidly increasing global population, coupled with growing food demands, evolving dietary preferences and the impact of climate change, pose considerable challenges in achieving widespread food and nutrition security [1, 2, 3]. In Australia, a country uniquely vulnerable to natural disasters, food production and consumption are major contributors to malnutrition, chronic disease and environmental degradation [4, 5]. The agricultural sector alone is responsible for 16% of Australia's greenhouse gas emissions [5], underscoring the imperative to address these environmental impacts within the food system. Recognising the ongoing susceptibility of food systems to climatic and global shifts, there is a pressing need for a transformative shift towards sustainability [6].

The 2030 Sustainable Development Goals highlight the importance of food systems in achieving global objectives and emphasise the need for nutrition professionals to become change agents in promoting sustainability [7, 8]. However, the current integration of sustainability into university nutrition and dietetics programmes is limited. For instance, a study found that fewer than 8% of Australian tertiary programmes included dedicated modules on sustainable food systems [9], raising concerns about whether the current curricula adequately prepare students for real‐world sustainability challenges [10, 11]. Despite updates to competency standards by Dietitians Australia [12] and the Nutrition Society of Australia [13], most education still focuses only on general nutrition and waste management rather than critically addressing the sustainability of dietary choices and food systems [9, 14, 15, 16]. The position statement of Dietitians Australia describes a sustainable food system as one that ‘provides nutritious, safe and culturally acceptable food while minimising environmental impact and promoting social equity {Barbour 2022 #45}’. Current nutrition and dietetics programmes in Australia have not widely embraced ‘systems thinking’ approaches when teaching students about food and nutrition through a sustainability lens [17].

Next‐generation solutions in dietetics require practitioners to think beyond individual behaviour change and work across policy, systems and environmental levels to address complex, adaptive challenges like climate change and food insecurity [18]. Systems thinking has been identified as a critical skill in the training of the future nutrition workforce, yet few curricula embed opportunities for students to develop these higher‐order thinking skills [19]. For example, a 2024 survey of US dietetics programme directors found that while most value systems thinking, only one‐third teach it, citing gaps in accreditation standards and limited awareness of available resources [20]. As a result, students may be underprepared to effectively incorporate a sustainability lens into their future professional practice. Within sustainability education, a systems approach allows students to view food beyond individual consumption and consider the interconnectedness of food production, processing, distribution and waste management [20]. Engaging students in food system assessments and urban agriculture initiatives can be particularly effective in illustrating these complex relationships [21, 22]. Urban agriculture, including community and home food production, has gained recognition as a key strategy for improving food security, environmental sustainability and public health [23]. Integrating urban agriculture into nutrition curricula may provide students with a tangible link between food production and dietary choices, reinforcing the importance of local and sustainable food systems, but this requires further investigation.

Effective education strategies are essential to prepare nutrition students for integrating sustainability into their professional practice [24]. However, most nutrition educators feel inadequately prepared to teach sustainability concepts, despite being interested in doing so [20, 25]. Traditional didactic methods, such as lectures and textbook‐based learning, are often used but may provide only foundational knowledge without fostering deep understanding or behaviour change around sustainability [9]. Experiential learning has been identified as a more effective approach, offering active engagement with real‐world applications of sustainability principles [26]. Indeed, a scoping review on sustainability education in nutrition and dietetics found that experiential learning activities were more successful in improving both knowledge and behaviour compared to passive learning methods [9]. Hands‐on learning experiences help students develop critical thinking skills, improve engagement and encourage meaningful reflection on how sustainability principles may apply to nutrition practice [8].

The aim of this study was to explore nutrition and dietetics students' knowledge, attitudes and behavioural intentions regarding sustainable and local food consumption; whether students' perceptions of sustainability impact their food procurement and purchasing behaviours; and whether these behaviours are associated with diet quality. Additionally, it evaluates the extent to which an experiential learning activity enhances awareness, knowledge and attitudes towards sustainable food systems, and whether it leads to increased intention to engage in sustainable food practices.

## 2 Materials and Methods

### 2.1 Study Setting

The study was conducted at the University of Wollongong, a tertiary institution established in 1975, located in the coastal city of Wollongong, New South Wales, Australia. The University of Wollongong campus has grown to encompass 10 additional campuses in Sydney and regional NSW along with numerous international partnerships [27]. As of 2023, there were a total of 25,813 onshore students enroled at the University of Wollongong, with approximately 80% studying at the University of Wollongong Campus [28]. In 2024, the university supported the introduction of an urban farming initiative incorporating an indoor aquaponic system (Farmwall Pty Ltd, herein referred to as ‘Farmwall’) on campus to promote environmental sustainability and address the food insecurity of students through the cultivation of locally grown microgreens [29]. Farmwall utilises aquaponics to cultivate microgreens and sprouts, offering a practical example of a low‐resource, high‐yield food production system that supports urban sustainability practices. The reason an aquaponic system was preferred over either hydroponic or soil‐based methods is that the Farmwall is a closed‐loop, self‐contained and compact unit that can easily be situated in an accessible place on campus for students to harvest their own microgreens for consumption. Additionally, the attractive drawcard of a fish tank provides visual appeal and may invoke curiosity and interest. A conventional soil‐based food garden would require much more intensive upkeep and may not expose the students to alternative ways of growing food in an urban environment.

### 2.2 Learning Activity

An experiential learning activity utilising Farmwall was developed by the research team. It was integrated into a module for public health nutrition subject, which is a 300‐level subject, typically taken in the third year of study. This subject is a core component of the undergraduate nutrition and dietetics programmes, but also includes postgraduate students completing equivalent coursework. The subject explores the intersection of food systems, public health and environmental sustainability.

The module includes a 2 h interactive lecture on sustainable food systems, a journal article reading on urban agriculture and a 2 h hands‐on tutorial engaging with the Farmwall system. Unlike previous years, where sustainability was introduced through didactic discussions, the redesigned tutorial was structured to immerse students in real‐world sustainability practices, providing them with a more interactive and inclusive learning experience. Kolb's Experiential Learning Theory (ELT) [30] was adopted to structure the Farmwall activity, guiding students through four stages: concrete experience, reflective observation, abstract conceptualisation and active experimentation. Students began with a concrete experience by individually tasting and identifying a variety of microgreens, then planting their own seeds to grow in the Farmwall aquaponic system. This hands‐on task introduced them to the sensory and practical aspects of local food production. In the reflective observation phase, students participated in small group discussions guided by prompts about the benefits and challenges of growing food locally, including considerations of access, sustainability and seasonality. These conversations allowed students to share diverse perspectives and reflect on their assumptions. During abstract conceptualisation, students worked in pairs or individually to connect their experiences with broader sustainability concepts, using structured worksheets to explore how local food systems relate to nutrition, environmental health and food equity. Students were encouraged to think about advantages (e.g., closed‐loop nature of water recycling; reusable growing pots; growing media; conversion of ammonia from fish excreta to nitrites and nitrates for plant nutrition, etc.) and potential disadvantages of an aquaponic system (e.g., barriers to upscaling; energy usage for lights and water pump, etc.). This allowed students to grasp the concept of an innovative circular food system. Finally, in the active experimentation phase, students applied their learning by individually creating visual communication resources (e.g., posters, infographics) that translated sustainability concepts into practical strategies for nutrition practice. The tutorial materials were designed to align with the subject curriculum, meeting key learning outcomes:

- Understanding the connections between food systems, environmental, societal and health concerns.
- Explaining the importance of the Planetary Health Diet in promoting sustainable, equitable and healthy food systems.
- Discussing the role of urban agriculture innovations in supporting sustainable diets.
- Identifying the role of dietitians and nutritionists in advancing sustainable food systems.

### 2.3 Study Design

A mixed‐methods embedded design was used to examine nutrition and dietetics students' knowledge, attitudes and behaviours towards sustainable and local food consumption. Using pre‐ and post‐surveys, quantitative data were collected to assess changes before and after the experiential learning activity. Qualitative data, in the form of open‐ended reflections embedded within the post‐survey, were used to explore students' experiences and intentions for behaviour change in greater depth. In this design, the qualitative insights complemented the primary quantitative findings, providing a more comprehensive understanding of the impact of the intervention. Ethical approval for the study was obtained from the Human Research Ethics Committee of the University of Wollongong (Ethics Number: 2024/024).

### 2.4 Participants and Data Collection

Data collection took place between 3 April 2024 and 21 April 2024. All students enroled in the Autumn 2024 core public health nutrition subject were invited to participate. An e‐invitation providing an overview of the project, learning activity and evaluation objectives was posted on the student online learning management system. Students who opted to participate accessed the corresponding pre‐activity survey via an online link and provided informed consent before proceeding. After completing the learning activity in their allocated tutorial sessions, students were provided with a link to complete the post‐activity survey.

### 2.5 Surveys

The survey questions were informed by existing tools and literature relevant to sustainability and food behaviours [31, 32], and were reviewed by the teaching team and student researcher for clarity and alignment with learning outcomes. The pre‐survey collected student demographic and education characteristics including age, gender, level of study, enrolment status, degree of enrolment and living situation. It also assessed students' perceptions and behaviours related to sustainable food choices using questions adapted from a previous study [31]. Using a five‐point Likert scale ranging from ‘not important’ to ‘very important,’ students were asked to rate the importance of purchasing foods and drinks with minimal environmental impact and the significance of various sustainable food procurement behaviours. These behaviours included growing their own food, finding sustainable foods affordable, purchasing locally grown, seasonal, organic or ethically certified foods, preferring minimal packaging and aiming for a more plant‐based diet. Additionally, students responded to yes‐or‐no questions regarding their participation in these specific food procurement behaviours. Students also self‐reported the frequency of making food purchasing choices aimed at minimising environmental impact (never, sometimes, about half the time, most of the time or always). Furthermore, students were surveyed on their knowledge and perceived importance of local food systems and their definitions of it, with response options including food produced within their region, within 100 kilometres, within their state or not sure. They were also asked about their views on campus initiatives for local and sustainable food practices, their interest in learning more about local food systems and sustainable agricultural practices and the perceived impact of choosing locally grown foods on the environment. Diet quality was only collected in the pre‐activity survey and evaluated using a validated food‐based questionnaire known as the Australian Recommended Food Score (ARFS) [32], which compares alignment of usual dietary patterns with the Australian Guide to Healthy Eating [33]. The questionnaire comprises 20 questions related to vegetables, 12 on fruits, 12 on breads and cereals, 10 on dairy foods, 7 on meat, 6 on meat alternatives, 2 on spreads or sauces and 1 on water intake. Scores range from 0 to 73, categorised as ‘needs work’ ( < 33), ‘getting there’ (33–38), ‘excellent’ (39–46) or ‘outstanding’ (47+).

In the post‐activity survey, the same questions from the pre‐activity survey were used to measure changes in students' perceptions, awareness and knowledge of local food systems, as well as their attitudes and intentions towards sustainability and local food consumption following the learning activity. Intention for change was measured using a five‐point Likert scale, ranging from ‘extremely unlikely’ to ‘extremely likely,’ where students rated their likelihood of adopting the same sustainable food procurement and purchasing behaviours covered in the pre‐activity survey. Additionally, this Likert scale was used to assess students' likelihood of applying their learnings in future nutrition/dietetic practice. Finally, students were asked two open‐ended questions about their key learnings from the experiential learning activity and suggestions for improving the activity for future learning experiences.

### 2.6 Data Analysis

Survey data were exported into IBM SPSS Statistics (Version 28) for analysis, with a significance level of 0.05 applied to all tests. Descriptive statistics were used to summarise demographic data, perceptions, awareness and knowledge of local food systems, and attitudes towards sustainable and local food consumption. To assess the baseline relationship between perceived importance and engagement in sustainable behaviours, five‐point Likert scale responses were recoded into binary categories (high vs. low importance), and Chi‐Square tests were conducted. Linear regression models examined differences in diet quality (ARFS scores) based on engagement in sustainable food procurement behaviours. For baseline and post‐activity comparisons, McNemar−Bowker tests assessed significant shifts in the proportion of students reporting high knowledge and positive perceptions regarding local food systems and university sustainability initiatives, as well as changes in the likelihood of adopting sustainable food behaviours.

Qualitative data on key learnings and suggestions for improvement were exported to an Excel spreadsheet on a password‐protected computer, deidentified, and analysed. A thematic analysis approach, as outlined by Braun and Clarke [34], was conducted to generate and review initial themes, then define and name the final themes before documenting the findings. Two members of the research team (K.K. and I.C.) independently reviewed and coded the qualitative responses. They met to discuss and reach a consensus on theme development, ensuring consistency in interpretation. Although formal inter‐rater reliability statistics were not calculated, the coding process was guided by reflexive discussion and cross‐checking of data to enhance rigour.

## 3 Results

All students enroled in the public health nutrition subject (*n* = 68) were invited to participate in the study. A total of 58 students completed both the pre‐ and post‐surveys, resulting in a response rate of approximately 85%. As shown in Table 1, most participants were female (87.9%), aged 18−24 years (70.7%) and domestic students (86.2%). The majority lived with family (63.8%) and were enroled in dietetics (44.8%) or nutrition (41.4%) programmes.

**Table 1:** Sociodemographic characteristics of the sample of university student respondents (*n* = 58).

Characteristic	Category	*n* (%)
Age	18−24	41 (70.7)
	25−35	12 (20.7)
	35+	5 (8.6)
Gender	Male	6 (10.3)
	Female	51 (87.9)
	Prefer not to say	1 (1.7)
Level of study	Undergraduate	51 (87.9)
	Postgraduate	7 (12.1)
Enrolment status	Domestic	50 (86.2)
	International	8 (13.8)
Degree of enrolment	Bachelor of nutrition and dietetics (honours)	26 (44.8)
	Bachelor of nutrition science	24 (41.4)
	Master of nutrition and dietetics	4 (6.9)
	Master of public health extension	3 (5.2)
	Bachelor of nutrition science and bachelor of psychology	1 (1.7)
Living situation	With family	37 (63.8)
	Off‐campus housing (not with family)	17 (29.3)
	On‐campus housing	3 (5.2)
	Other	1 (1.7)

### 3.1 Baseline Perceptions and Behaviours

At baseline, students reported strong support for local food systems, with 77.6% believing they are very important and 70.7% defining local food as being regionally produced (Table 2). However, only 5.2% felt they had strong knowledge, indicating a knowledge gap. While 60.3% see a significant positive impact of locally grown food, fewer (43.1%) were very interested in learning more. Additionally, only 39.7% agreed that their university supports local and sustainable food practices.

**Table 2:** Baseline perceptions of local food systems among university students (*n* = 58).

	% (*n* = )
Local food is food produced within the region (% agree)	70.7% (41)
Strong knowledge of local food systems (% self‐reported strong knowledge)	5.2% (3)
Local food systems are very important (% agree)	77.6% (45)
Significant positive impact of locally grown foods (% agree)	60.3% (35)
Very interested in learning about local food systems and sustainable agricultural practices (% agree)	43.1% (25)
Agree that the university supports local and sustainable food practices on campus (% agree)	39.7% (23)

*Note:* Items reflect Likert‐type responses. Results are reported as the percentage of students selecting the most positive response option (e.g., strongly agree, very important or very interested), depending on the wording of each item.

Table 3 presents both students' perceived importance (measured using a five‐point Likert scale) and actual engagement (measured via yes/no questions) in specific sustainable food behaviours. Despite this, the majority placed high importance on sustainable food practices (see Table 3), particularly purchasing foods and drinks with minimal packaging (69.0%), buying locally grown foods (60.3%) and purchasing ethically certified products (56.9%). Conversely, the least valued behaviours were buying mostly plant‐based foods (27.6%) and purchasing organic foods (31.0%).

**Table 3:** Relationship between perceived importance (perception) and engagement (behaviour) in sustainable food practices among university students (*n* = 58).

Food procurement and purchasing behaviours	Rated as high importance (perception) *n* (%)	Engaged in behaviour (yes) *n* (%)	Chi‐square value	*p* value
Growing own food	22 (37.9%)	24 (41.4%)	7.239	0.007
Buying locally grown	35 (60.3%)	17 (29.3%)	4.868	0.027
Buying seasonal foods	29 (50.0%)	44 (75.9%)	9.416	0.002
Buying organic foods	18 (31.0%)	12 (20.7%)	8.976	0.003
Buying ethically certified	33 (56.9%)	28 (48.3%)	7.234	0.007
Buying with minimal packaging	40 (69.0%)	36 (62.1%)	5.957	0.015
Buying mostly plant‐based foods	16 (27.6%)	20 (34.5%)	11.484	< 0.001

Regarding engagement in sustainable food practices, students most frequently reported buying seasonal foods (75.9%) and purchasing food and drinks with minimal packaging (62.1%). However, participation in other sustainable behaviours was relatively low, with less than a third purchasing locally grown foods and fewer than a quarter buying organic foods. Chi‐square tests were used to assess whether students who rated each behaviour as highly important were also more likely to report engaging in that behaviour. The strongest associations were observed for buying mostly plant‐based foods and buying seasonal foods, suggesting that valuing these practices significantly predicts participation. The weakest association was found for buying locally grown foods indicating that while many students value this behaviour, fewer actively engage in it (Table 3).

High importance reflects students who rated each behaviour as important or very important. Engagement reflects those who answered ‘yes’ to participating in the behaviour.

The average diet quality score among students was 39.7 ± 8.4 ARFS, classified as ‘excellent’. Vegetarian students had a slightly higher diet quality score (43.0 ± 3.7) compared to non‐vegetarians (39.3 ± 8.7). Linear regression analysis demonstrated significant associations between sustainable food practices and diet quality, with growing food, buying locally grown foods and purchasing seasonal foods linked to an average increase of approximately five ARFS points (Table 4).

**Table 4:** Summary of linear regression tests for the relationship between food procurement and purchasing behaviours and ARFS scores in 3rd‐year students (*n* = 58).

Predictor variable	Coefficient	SE	*t*‐value	*p* value
Growing own food	4.439	2.187	2.029	0.047
Buying locally grown	5.265	2.349	2.242	0.029
Buying seasonal foods	5.899	2.486	2.373	0.021
Buying organic foods	0.812	2.753	0.295	0.769
Buying ethically certified	4.260	0.255	1.972	0.054
Buying with minimal packaging	4.114	2.234	1.842	0.071
Buying mostly plant‐based foods	3.145	2.310	1.361	0.179

### 3.2 Post‐Learning Activity

**Figure 1:**
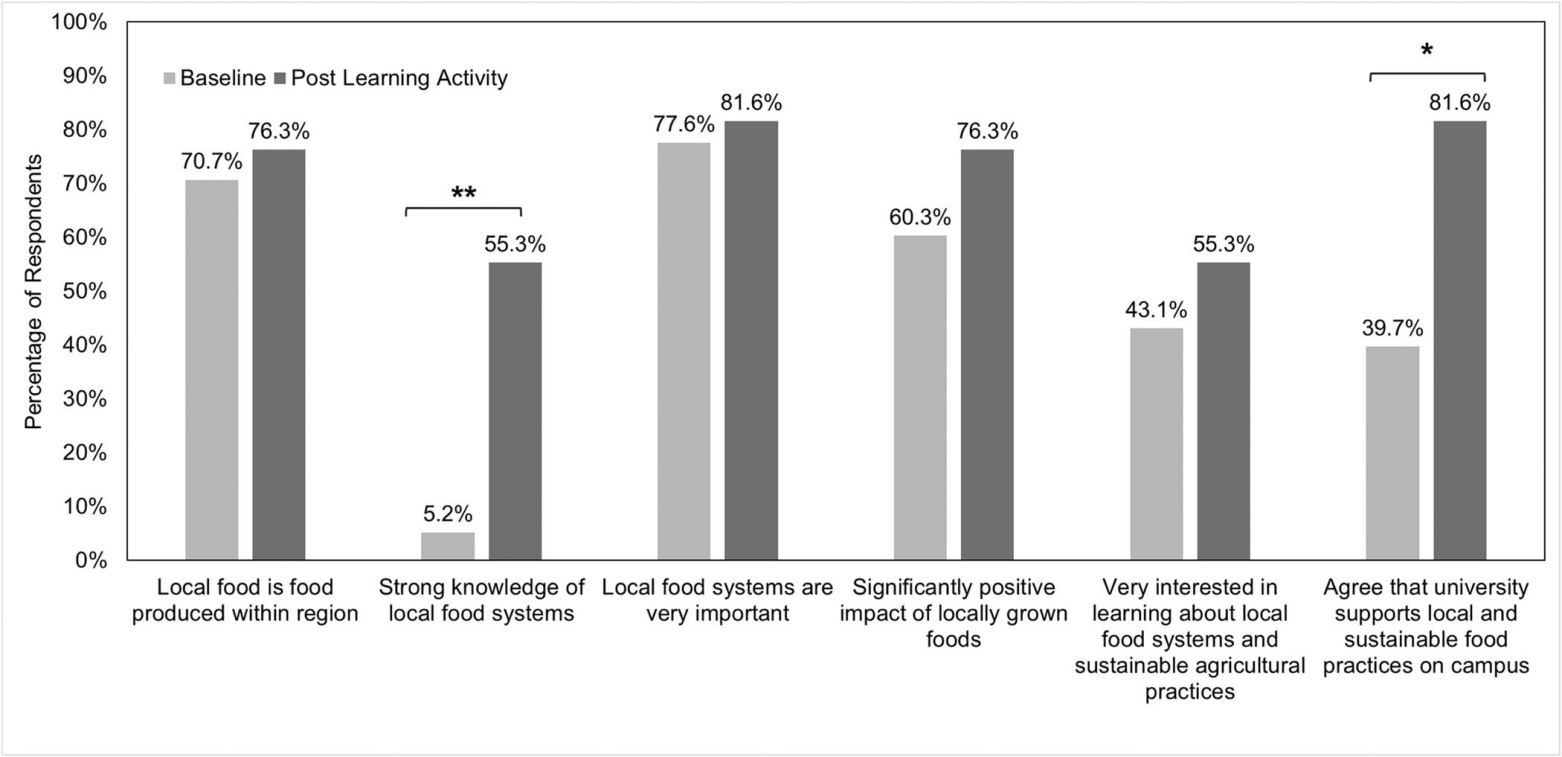
Comparison of baseline and post‐activity responses for knowledge and positive attitudes towards local food systems and sustainability (*n* = 58). *p < 0.05, **p < 0.001, *p*‐value derived from McNemar−Bowker tests.

Participation in the module and experiential learning activity resulted in a significantly larger proportion of students reported high knowledge of local food systems and expressed agreement with the universities support of local and sustainable food practices on campus (Figure 1).

**Figure 2:**
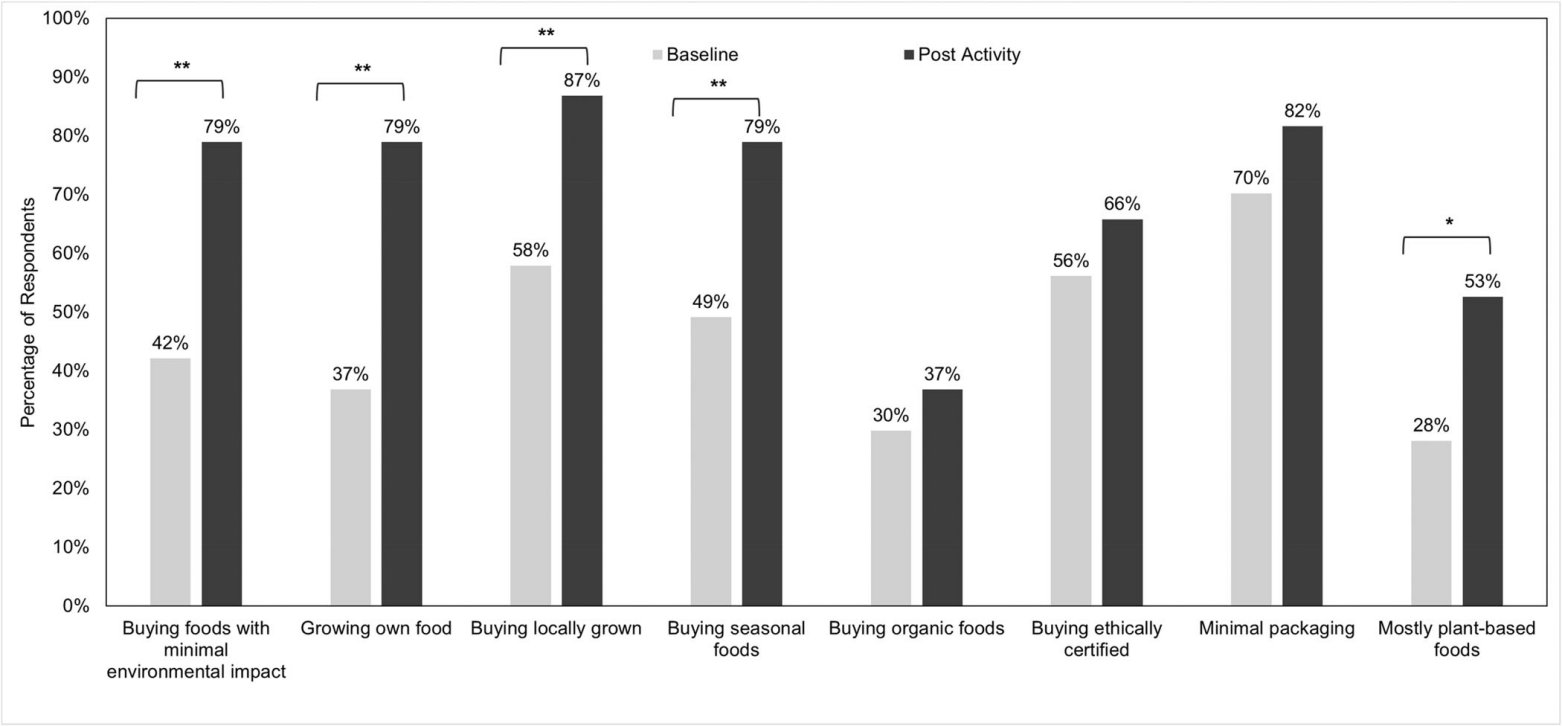
Comparison of baseline and post‐activity responses for intentions towards adopting sustainable dietary behaviours (*n* = 58). *p < 0.05, **p < 0.001, *p*‐value derived from McNemar−Bowker tests.

Most participants reported that they would implement learnings from the tutorial activity into future practice (81.6%). After the tutorial, there was a significant increase in the proportion of students who reported intentions to adopt various food procurement behaviours (Figure 2), including purchasing foods with minimal environmental impact, growing their own food, buying locally grown foods (all *p* < 0.001) and eating mostly plant‐based foods (*p* < 0.05).

Thematic analysis of open‐ended responses identified four themes including [1]: The complexity of sustainability within food systems [2]; the identification and advantages of sustainable practices [3]; promoting healthy and sustainable diets; and [4] the incorporation into diverse professional practice. The responses highlighted a clear understanding for the intricate challenges of sustainability within food systems, with several recognising that food systems involve interconnected environmental, social and economic dimensions. One student remarked on learning about ‘how closely knit our food systems are with the overall health of ourselves and also the environment around us’ (Participant 2). They also consistently recognised the learning activities' focus on the environmental benefits of reducing food transportation, and practices such as promoting locally grown and urban agriculture. Students identified that foods produced this way could be nutrient‐dense food options and integral to sustainable diets. For instance, one participant highlighted they learned the importance of ‘incorporating highly nutrient and sustainable foods sourced locally to minimise transport and packaging’ (Participant 18). Others highlighted they learned more about the benefits of ‘encouraging local produce as a healthy habit’ (Participant 6). Another student explained they learned more about the link between dietary choices, health and environmental impact: ‘growing your own little plants can attribute to big health benefits’ (Participant 12). Furthermore, participants considered the future of sustainability and its broad influence on personal and professional lives. For instance, one participant reflected on the bigger role of sustainability in food systems and that ‘sustainable food systems will help future food industry’ (Participant 30), underscoring the transformative potential of sustainable practices. Another participant highlighted they learned the importance of ‘promoting sustainably grown produce’ (Participant 23) as a nutrition professional, indicating a commitment to fostering environmentally responsible agricultural practices.

Participants were also asked to provide feedback on how the tutorial and activity could be enhanced for future use. Three key recommendations were derived [1]: sustained engagement across the curriculum [2]; increased awareness and outreach; and [3] broad interdisciplinary participation. Feedback from participants underscored a desire for more interactive and sustained engagement with the Farmwall unit throughout the semester. One participant suggested, ‘make it a more interactive thing throughout the semester. I probably won′t really think about this again unless it′s another tutorial in class’ (Participant 1), emphasising the need for continuous involvement beyond initial interactions. Others recommended expanding its presence across campus, stating, ‘add it to other cafes or food services around campus’ (Participant 2), and allocating dedicated times for student visits outside of tutorial hours. Enhancing awareness was also a key theme, with suggestions like, ‘creating a strong and engaging social media platform’ (Participant 7) and ‘engaging more classes for larger distribution of information’ (Participant 8). Participants highlighted the value of regular activities such as maintenance, harvests and fish feeding to sustain interest and deepen learning experiences. They also expressed an interest to involve more faculties and students in Farmwall activities to broaden its impact on campus with one participant stating that we should be ‘encouraging more faculties to get involved’ (Participant 26).

## 4 Discussion

This study demonstrated that third‐year nutrition and dietetics university students recognised the importance of sustainable food practices; however their baseline knowledge of local food systems was low and their level of engagement in adopting sustainable food behaviours varied according to their underlying values. Students who participated in sustainable food practices had higher diet quality scores which may suggest that they were more focused on food generally, both from a human health and an environmental point of view. These findings indicate a need for ongoing integration of systems thinking throughout the curriculum to effectively translate knowledge gains into sustained and practical behaviour changes.

Current literature highlights a gap in sustainability education within Australian nutrition programmes, potentially leaving students underprepared for real‐world challenges after graduation [9]. Our results contribute to this literature, suggesting that embedding experiential learning may help bridge this gap. Similar to our findings, another Australian study found that while students generally hold positive attitudes towards sustainable food choices, many lack comprehensive knowledge about the sustainability of the food system overall [10]. Our study also aligns with previous research indicating that the perceived importance of sustainable behaviours translates into greater engagement among students [35]. We found that nutrition students place value in purchasing foods and drinks with minimal packaging which contrasts a large survey where less than half of Australians aged over 18 years were concerned about food packaging in their purchasing decisions [36]. This discrepancy may highlight a generational difference, with younger populations showing a stronger environmental consciousness [37]. Previous research also identifies the consumption of locally grown foods as a prevalent environmentally‐conscious practice amongst Australians [38]. Our study builds on this by demonstrating positive attitudes towards locally grown food among students. However, the gap between positive attitudes and lower reported purchasing of locally grown foods may be attributed to the perceived higher cost, a barrier previously identified among Australian adults [39]. The reported engagement with buying seasonal foods aligns with this barrier of cost, as seasonal items tend to be more affordable [39]. Interestingly, our study found that plant‐based foods were neither highly valued nor frequently purchased by students, which could be attributed to limited exploration of these options within sustainability education. Alternatively, this pattern may reflect a broader trend among younger Australians who remain relatively unreceptive to plant‐based options due to concerns about taste [40] and perceived availability and accessibility [36]. Additionally, our results align with the understanding that sustainable practices, as outlined in the EAT Lancet Planetary Health Diet, correlate with better diet quality [41], as evidenced by the higher diet quality scores among students who engaged in more sustainable behaviours. Additionally, emerging research suggests that nutrition and dietetics students may hold more pro‐environmental attitudes than their peers in other disciplines. For instance, Burkhart et al. found that most Australian nutrition and dietetics students viewed sustainability as very important to both society and their future professional roles, with particularly high familiarity and concern for environmental sustainability [10]. This may partly explain the relatively high engagement with sustainable food behaviours observed in our cohort, even in the absence of comprehensive sustainability education.

Participation in the experiential learning activity utilising a novel, indoor alternative method to growing food using an aquaponic system led to notable improvements in students' reported knowledge, attitudes and perceptions, as well as intentions regarding behaviour change. These positive outcomes were anticipated given previous studies that have demonstrated the effectiveness of experiential learning in enhancing knowledge, skills and behaviours [8]. The experiential learning activity itself emerged as a powerful tool for stimulating reflection and building practical understanding. While the integration of urban agriculture in nutrition education is unusual in Australia, a similar Canadian study using community gardens within a nutrition course achieved comparable benefits, particularly in terms of acquiring course content and food literacy skills [42], suggesting that hands‐on learning methods are broadly effective. Additionally, research from the US highlights that integrating urban agriculture in undergraduate biology courses provides tangible benefits for developing core competencies [22]. Students in our study expressed a desire for ongoing engagement throughout the semester, greater community involvement and increased campus awareness. Participants also identified key learnings, particularly highlighting the complexity of sustainability—a theme consistently noted among nutrition students in other studies [43]. Interestingly, educators also identify teaching this complexity as a challenge in nutrition and dietetics education, reinforcing the need for innovative approaches to build systems thinking capabilities among students [20]. Importantly, some students demonstrated an emerging systems perspective, recognising the interconnectedness between food production, environmental impact, health outcomes and community resilience. This suggests that experiential learning can serve as an entry point for introducing systems thinking—an essential competency for navigating the complexity of sustainability in food systems [20]. Embedding systems thinking alongside experiential activities may strengthen students' capacity to critically evaluate food system challenges, identify leverage points for change and develop holistic, practical solutions as future practitioners. While our study has demonstrated that experiential learning activities using Farmwall enhance students' understanding and intentions towards sustainability, it is still essential to continue exploring innovative educational strategies to better equip students to address these challenges.

The results of our study should be considered in the context of some limitations including the use of convenience sampling and a small sample size, which limit generalisability to broader populations. The predominance of female participants in this study reflects the national demographic profile of nutrition and dietetics students in Australia, where enrolments are overwhelmingly female [43]. However, this may limit the generalisability of findings to other student populations or disciplines with more gender‐diverse cohorts. Additionally, reliance on a non‐validated questionnaire for sustainability‐related knowledge, attitudes and behaviours may have introduced potential measurement biases, while the short‐term study design prevents assessment of long‐term behaviour change. Self‐reported dietary data and data on sustainable behaviours may also introduce measurement bias. Future research should involve larger, more diverse samples and consider longitudinal studies to assess the enduring effects of experiential learning on sustainable behaviours. Future research should also explore how experiential learning and systems thinking can be embedded longitudinally across programmes and evaluated for sustained impact.

## 5 Conclusion

Our study findings demonstrate a gap between sustainability knowledge and its practical application among nutrition and dietetics students; however, the use of an indoor aquaponic system as an experiential learning tool shows promise in bridging this gap, suggesting that such hands‐on activities can help translate knowledge into actionable behaviours. Given that only intention to change was measured, sustained engagement with experiential learning throughout the curriculum should be considered to foster long‐standing behaviour change. Furthermore, the finding that students who engage in sustainable practices exhibit better diet quality underscores the value of integrating sustainability education into nutrition courses. Universities should provide more experiential learning opportunities across faculties to advance sustainability education and equipping students to tackle real‐world challenges in promoting sustainable diets.

## Author Contributions

Karen E. Charlton and Anne‐Therese McMahon conceptualised the study. All authors contributed to the development of the study methodology. Katherine Kent and Isabelle Crowe designed the survey and managed data analysis, with input from all authors on data interpretation. Isabelle Crowe drafted the manuscript, while Katherine Kent and Karen E. Charlton provided input on the introduction, methods, results and abstract. Katherine Kent, Karen E. Charlton and Anne‐Therese McMahon reviewed and approved the final version. The authors acknowledge Farmwall for their contribution to the conceptualisation and delivery of the learning activity.

## Ethics Statement

Ethical approval for the study was obtained from the Human Research Ethics Committee of the University of Wollongong (Ethics Number: 2024/024).

## Conflicts of Interest

Indiana Rhind is a former employee of Farmwall. Indiana helped to design the experiential learning activity but was not involved in the evaluation data collection, analysis or interpretation of this evaluation. There is no direct financial benefit for her involvement in this evaluation. The other authors declare no conflicts of interest.

## 1 Peer Review

The peer review history for this article is available at https://www.webofscience.com/api/gateway/wos/peer-review/10.1111/jhn.70103.

## Data Availability

The data that support the findings of this study are available from the corresponding author upon reasonable request.
